# Meta-analysis of Molteno glaucoma implants and Ahmed glaucoma valves: insights into efficacy and safety for complex glaucoma

**DOI:** 10.3389/fopht.2023.1307439

**Published:** 2023-12-11

**Authors:** Adarsh Raja, Sunaina Bhimani, Hafsah Alim Ur Rahman, Madiha Salman, Afrah Saeed Shaikh, Sandesh Raja, Bushra Zafar Sayeed, Ahsan Feroze, Muhammad Ahmed

**Affiliations:** ^1^ Department of Medicine, Shaheed Mohtarma Benazir Bhutto Medical College Lyari, Karachi, Pakistan; ^2^ Department of Medicine, Dow University of Health Sciences, Karachi, Pakistan; ^3^ Ziauddin Medical College, Ziauddin University, Karachi, Pakistan; ^4^ Department of Medicine, Aga Khan University Hospital, Karachi, Pakistan; ^5^ Department of Medicine, Jinnah Postgraduate Medical Centre, Karachi, Pakistan

**Keywords:** glaucoma, Molteno glaucoma implant, Ahmed glaucoma valve, surgery, glaucoma—surgery

## Abstract

**Background:**

Glaucoma is a leading cause of irreversible blindness globally and for decades, Molteno and Ahmed glaucoma implants, operating on different mechanisms, have been used to treat complicated glaucoma with varying success.

**Objective:**

To assess the safety and efficacy of the Molteno glaucoma implant (MGI) versus the Ahmed glaucoma valve (AGV) in patients with complicated glaucoma.

**Methods:**

We comprehensively searched PubMed, Google Scholar, Cochrane Library and Science Direct) from inception till July 2023 and studies comparing patients with MGI and those with AGV in patients with complicated glaucoma. The primary outcome was intra-ocular pressure reduction at different time intervals. Secondary outcomes included surgical success rate, hypertensive phase, anti-glaucoma medication (AGM) and total complications.

**Results:**

In this meta-analysis, four studies were included with a patient population of 257 with refractory, neovascular or advanced uncontrolled glaucoma. Postoperative intra-ocular pressure reduction did not show significant difference between the two groups (MD: -1.34, 95% CI [-2.78, 0.09]). From the secondary outcomes, surgical success rate (RR: 0.88, 95% CI [0.51,1.53]), hypertensive phase (RR: 0.74, 95% CI [0.39,1.40]) were insignificant. Postoperative anti-glaucoma medication (MD: -0.07, 95% CI [-0.79, -0.65] and total complications (RR:1.36, 95% CI [1.07, 1.72]) were significant.

**Conclusion:**

No significant difference was observed between the patients with MGI and AGV for the primary outcome. From the secondary outcome, AGV was associated with reduced anti-glaucoma medication use and significantly lowered the number of complications.

**Systematic review registration:**

https://www.crd.york.ac.uk/prospero/display_record.php?RecordID=475539, identifier CRD42023475539.

## Introduction

A series of vision problems known as glaucoma are characterized by elevated intraocular pressure (IOP) and a progressive degeneration of retinal ganglion cells. Glaucoma can be identified by the progressive loss of peripheral vision, which comes first, then the loss of central vision. Without proper treatment, glaucoma can cause total blindness (1). Glaucoma is the second-leading cause of blindness in the world, after cataracts. In 2020, glaucoma accounted for 11% of all cases of adult blindness in the world among those 50 years and older (2). Due to the high risk of failure with conventional filtration surgery, glaucoma drainage devices (GDDs) are now frequently used in the treatment of refractory glaucoma (3). This includes eyes that have undergone trabeculectomy or other eye surgery that left conjunctival scarring, and eyes with secondary glaucoma such as post-keratoplasty, neovascular, or traumatic glaucoma, which are known to have poor outcomes with trabeculectomy (4). Aqueous drainage devices have a high level of efficacy as a first-line surgical therapy despite the high-risk profile of these patients (5). Over the past two decades, a variety of glaucoma drainage implants have been created (6). These include GDDs, including both valved and non-valved ones, such as the Ahmed glaucoma valve (AGV) (New World Medical, Rancho Cucamonga, CA, USA), the Molteno glaucoma implant (MGI) (Molteno Ophthalmic Ltd., Dunedin, New Zealand), the Baerveldt glaucoma implant (Advanced Medical Optics, Inc., Santa Ana, CA, USA), and the Aurolab aqueous drainage implant device (Aurolab, Madurai, India). (7) The AGV and double-plate Molteno are the aqueous drainage devices that are implanted most frequently (8). The AGV features a one-way valve that shields the anterior chambers from hypotony and shallowness after surgery. AGV implants have a reported success rate that ranges from 60% to 85%, depending on the length of the follow-up period (9, 10). However, in addition to the high cost of AGV implants, multiple studies have linked them to high rates of encapsulation and insufficient IOP reduction, necessitating the use of glaucoma medicines after surgery (8, 11). The Molteno implant was the initial glaucoma draining device. In 1969, this GDD was first administered, presenting the fundamental concept on which all current GDDs are built (12). A silicon tube linked to a tube inserted 9 mm–10 mm posterior to the limbus in the subconjunctival area makes up the Molteno implant, a non-valvular device. The amount of aqueous drainage and the ultimate IOP are influenced by the surface area of the fibrovascular, permeable bleb that forms over the Molteno implant plate after it is sutured to the sclera and covered by tenon tissue and conjunctiva (12). In refractory glaucoma, the reported success rates with double-plate Molteno range from 62% to 95% (13). There are now several studies being conducted that compare these two types of glaucoma drainage implants for the treatment of refractory glaucoma. As each implant has a unique set of advantages and disadvantages, it is impossible to draw firm conclusions about differences in the findings of objective tests. To the best of our knowledge, comparisons of the effectiveness and safety of these two techniques have not been systematically evaluated and published. We therefore performed a literature-based meta-analysis to compare the efficacy and safety of the MGI with the AGV in the treatment of complex glaucoma and to determine the best course of action for complex glaucoma. This meta-analysis examines the two glaucoma implants made by Molteno and Ahmed, providing answers to important clinical queries and enhancing patient outcomes.

## Materials and methods

This systematic review and meta-analysis was conducted according to the Preferred Reporting Items for Systematic Reviews and Meta-Analyses (PRISMA) guidelines (14).

### Data sources and search strategy

A comprehensive literature search was conducted on PubMed (National Library of Medicine, Bethesda, MD, USA), Google Scholar (Google Scholar Google Inc., Mountain View, CA, USA), the Cochrane Library, and ScienceDirect (Elsevier, Amsterdam, the Netherlands) from inception to July 2023. Online databases such as www.clinicaltrials.gov and medRxiv.org, and conference proceedings and presentations were also searched to identify gray literature. The bibliographies of relevant articles were also searched to make sure that no studies were missed. No restrictions on language or publication date were imposed. The detailed search strategies used in these databases are provided in **Supplementary Table 1**.

### Study selection

All articles retrieved from the databases were transferred to the EndNote X9 (Clarivate™, Philadelphia, PA, USA), to remove duplicate articles. Two independent investigators (B.Z.S. and H.A.U.R.) screened the remaining articles, based on title and abstract, and then full-text articles were reviewed. In case of any disagreement, a third reviewer (S.B.) was consulted. The studies were selected based on the following inclusion criteria: (a) the article was an original study, (b) the study compared AGV with MGI, and (c) outcomes of interest were reported, including IOP reduction, the success rate of the implant, hypertensive phase, and the reduction in antiglaucoma medication. The exclusion criteria consisted of (a) studies without a valid comparison group, that is, a type of glaucoma drainage implant other than MGI and AGV, (b) duplicate studies, and (c) outcomes of interest not being reported.

### Data extraction and quality assessment

Two investigators (A.R. and S.R.) independently extracted the data from the included studies into a Microsoft Excel (Microsoft Corporation, Redmond, WA, USA) sheet that was created. When there were discrepancies, a cooperative strategy was used to reach an agreement. To resolve disagreements over how to interpret the data, the two researchers had lengthy conversations. If a settlement could not be achieved, S.B. was consulted as an unbiased arbiter to offer a conclusive viewpoint. The following data were extracted from each study: (a) the study name and year of publication, (b) the study design, (c) the mean age of patients in each group, (d) the number of patients in each group (MGV vs AGV), (e) the length of follow-up period, and (f) all outcomes of interest. The quality assessment was performed using the Cochrane Risk of Bias (RoB 2.0) tool (15) for randomized controlled trials (RCTs) and the Newcastle–Ottawa Scale for observational studies (16). Despite employing standardized tools for quality assessment, the inherent subjectivity in interpretation or potential disagreements among reviewers were mitigated by consulting another researcher, which involved extensive discussions. These deliberations were aimed at fostering consensus among the investigators, ensuring a unified understanding and application of the assessment criteria.

### Statistical analysis

For the statistical analysis, ReviewManager (RevMan Version 5.4.1) (Cochrane Collaboration, London, UK) was used. A relative risk (RR) assessment with 95% confidence intervals (95% CIs) was performed for dichotomous outcomes, whereas the mean difference (MD) was used for continuous outcomes. All the results were reported with 95% CIs. A random-effects model was used to pool the outcomes, and the statistical heterogeneity was measured using Higgins’s *I* ^2^; the value of *I* ^2^ < 50% was considered mild heterogeneity, 50%–75% was considered moderate heterogeneity and > 75% was considered severe heterogeneity (17). A *p*-value of ≤ 0.05 was considered statistically significant. A leave-one-out analysis was performed for the outcomes with severe heterogeneity.

## Results

### Study selection and characteristics

**Figure 1:**
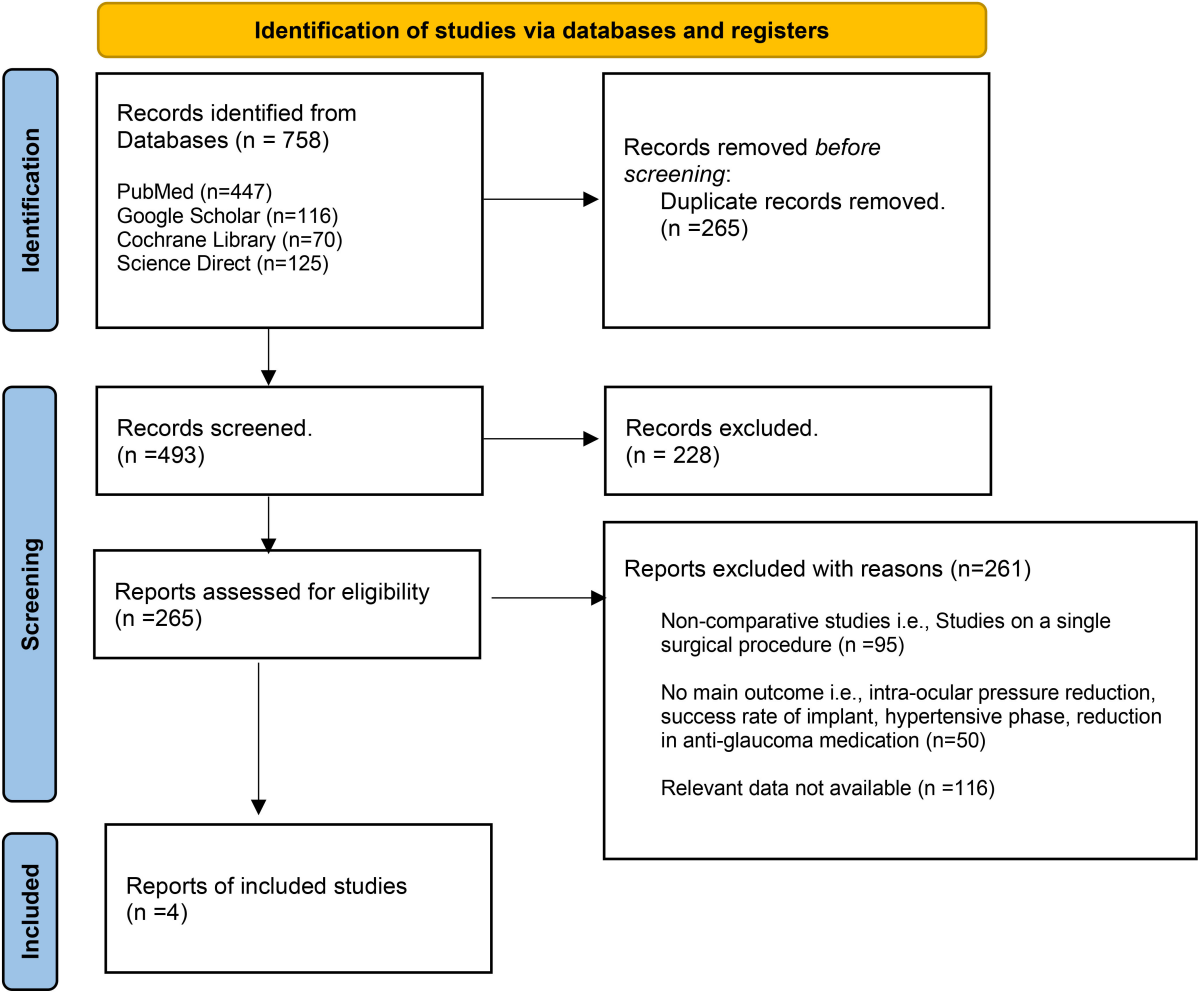
PRISMA flow chart.

A total of 758 articles were retrieved from all the electronic databases. After removing duplicates and assessing for eligibility, four articles were included in this meta-analysis (8, 18–20). Of the four studies, two were retrospective cohort studies (18, 19), one was a case–control study (8), and one was an RCT (20). The PRISMA flow chart (**Figure 1**) gives a summary of the findings of our literature search.

The number of patients from these four studies totaled 257 (130 with an MGI and 127 with an AGV). The mean age of patients was 63.2 years in the MGI arm and 62 years in the AGV group. The follow-up period of the included studies ranged from 20 months up to 41.9 months. The general characteristics of the included studies are summarized in **Table 1** and the baseline characteristics of the patients are presented in **Supplementary Table S2**. The quality assessment of the observational studies was performed using the Newcastle–Ottawa Scale, and all the included studies were rated as being of “high quality” (**Supplementary Tables S3** and **S4**). The details of the risk assessment are given in **Supplementary Figures S1** and **S2**.

**Table 1:** General characteristics of the included studies.

Study	Design	Year of publication	Country	Type of glaucoma	Study duration	Sample size (*n*)		Device type	
						MGI group	AGV group	MGI group	AGV group
Taglia et al.	Cohort study	2002	USA	Refractory	1 June 1991 to 1 October 1997	27	13	Double-plate Molteno	AGV Ns
Ayyala et al.	Case control	2002	USA	Advanced uncontrolled	January 1993 to June 1996	30	30	Double-plate Molteno	AGV Ns
Yalvac et al.	Cohort study	2005	Turkey	Neovascular	May 1997 to May 2002	27	38	Single-plate Molteno	AGV model S-2
Nassiri et al.	Randomized trial	2010	Iran	Refractory	January 2003 to August 2005	46	46	Single-plate Molteno	AGV model FP7

Ns, not specified.

### Primary outcome

#### Postoperative intraocular pressure

**Figure 2:**
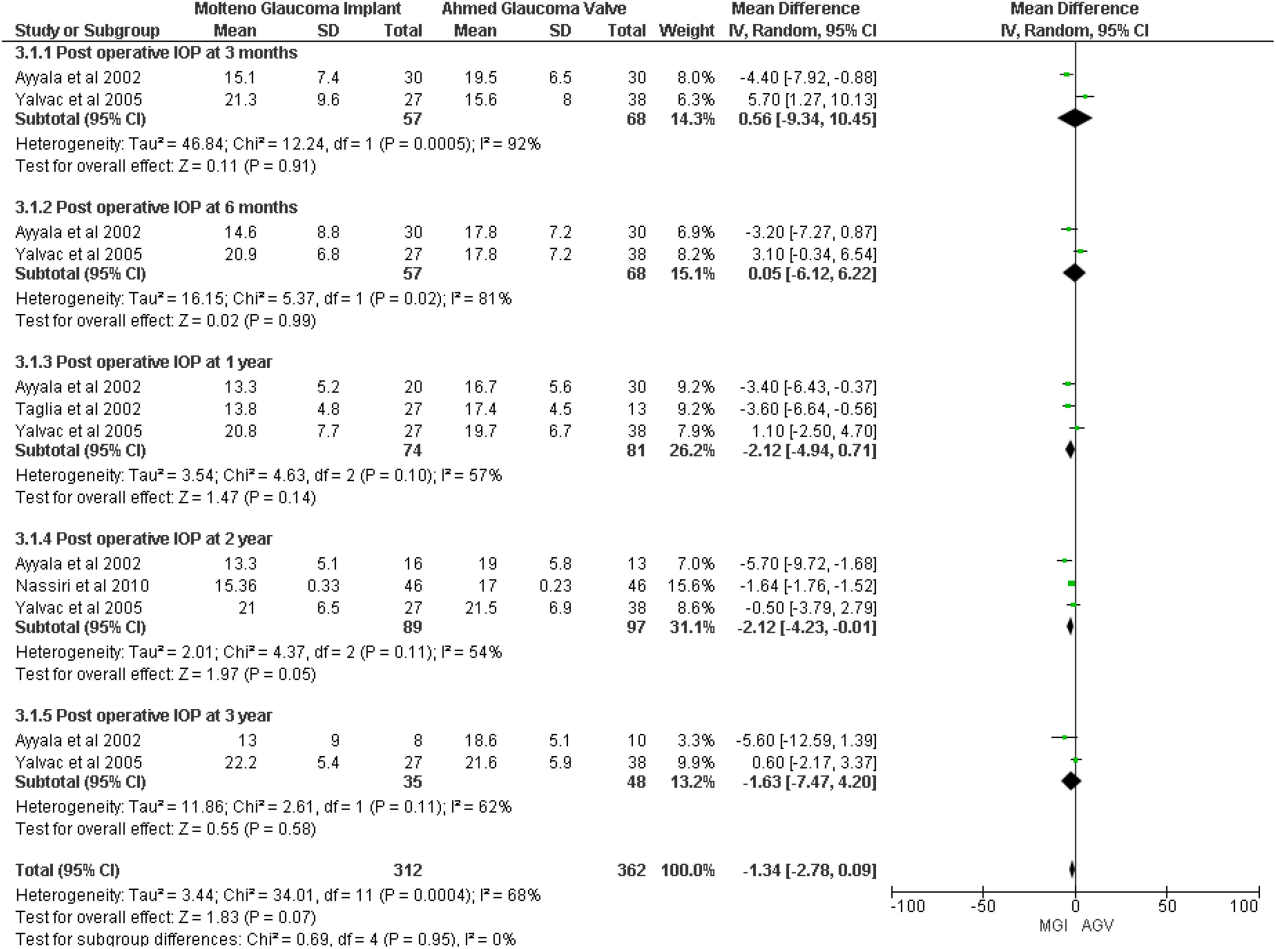
Forest plot of intraocular pressure at different time intervals.

A meta-analysis of four studies (8, 18–20) showed that no significant difference was observed between the MGI and AGV groups in the outcome of postoperative IOP (MD −1.34, 95% CI −2.78 to 0.09; *p* = 0.07; *I* ^2^ = 68%). However, IOP at 2 years showed a significant difference in the MGI group (MD −2.12, 95% CI −4.23 to −0.01; *p* = 0.05; *I* ^2^ = 54%) (**Figure 2**).

### Secondary outcomes

#### Surgical success rate

**Figure 3:**
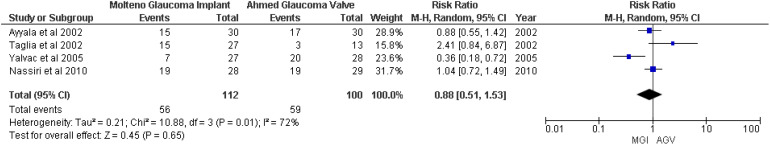
Forest plot of surgical success rate.

Surgical success was defined as an IOP < 22 mmHg and > 5 mmHg without additional glaucoma surgery and without loss of light perception (18). A random-effects meta-analysis of four studies (8, 18–20) showed no significant association between the MGI group and the AGV group (RR 0.88, 95% CI 0.51 to 1.53; *p* = 0.65; *I* ^2^ = 72%) (**Figure 3**).

#### Hypertensive phase

**Figure 4:**
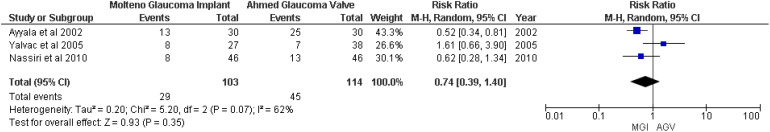
Forest plot of hypertensive phase.

For the outcome hypertensive phase, no significant association between the MGI and AGV groups was found (RR 0.74; 95% CI 0.39 to 1.40; *p* = 0.35; *I* ^2^ = 62%) (**Figure 4**).

#### Postoperative antiglaucoma medication

**Figure 5:**
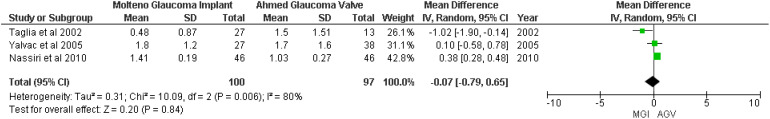
Forest plot of postoperative antiglaucoma medication.

Three studies (18–20) were included in the random-effects meta-analysis of postoperative antiglaucoma medication. The pooled result showed that there was no significant difference between the MGI and AGV groups (MD −0.07; 95% CI −0.79 to −0.65; *p* = 0.84; *I* ^2^ = 80%) (**Figure 5**).

#### Total complications

**Figure 6:**
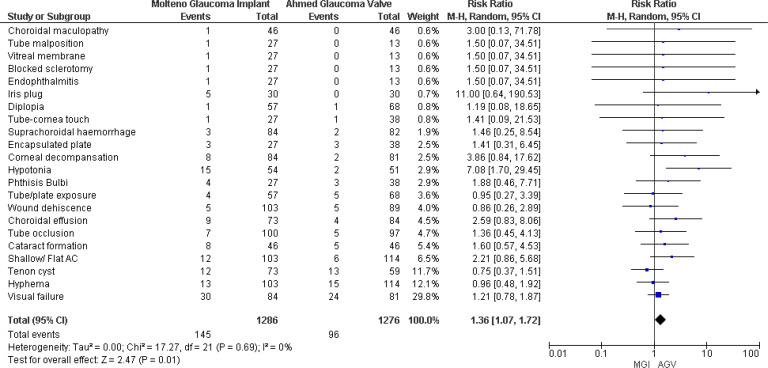
Forest plot of total complications.

Four studies (8, 18–20) reported postoperative complications, and pooled results showed that patients in the MGI group experienced more complications than those in the AGV group. The results were statistically significant, and no significant heterogeneity was observed (RR 1.36, 95% CI 1.07 to 1.72; *p* = 0.01; *I* ^2^ = 0) (**Figure 6**).

### Assessment of heterogeneity

To ensure the pooled estimates’ robustness and to prevent disproportionate impacts, a sensitivity analysis was conducted, which involved excluding studies with small or large sample sizes and outliers. For the outcome of IOP, the removal of Yalvac et al. from the 3-month and 6-month intervals demonstrated that there was a significant difference, with a significant decrease in heterogeneity (MD 2.14, 95% CI −3.32 to −0.96; *p* = 0.00004; *I* ^2^ = 44%) (**Figure S3**). In the outcome of surgical success rate, the exclusion of Yalvac et al. showed a decrease in heterogeneity from *I* ^2^ = 72% to *I* ^2^ = 36% (**Figure S4**). The removal of Yalvac et al. from the outcome of hypertensive phase outcome resulted in a reduction of the *I* ^2^ value to 0% from 62% (**Figure S5**). The removal of Taglia et al. from the outcome of antiglaucoma medication decreased heterogeneity to *I* ^2^ = 0% from *I* ^2^ = 80%, which showed that it was heavily influenced by this study (**Figure S6**).

## Discussion

The efficacy and safety of two glaucoma drainage implants, the MGI and the AGV, have been compared in the current meta-analysis by reviewing the body of available literature. The Molteno implant was historically the first device used to drain glaucoma. It is a non-valvular device composed of a silicon tube, and its efficiency is dependent on the surface area of the plates, which contributes to the amount of aqueous drainage and final IOP. In contrast, the Ahmed glaucoma implant consists of three parts: a plate made of polypropylene, silicone, or polyethylene; a silicone drainage tube; and a silicon valve. As it is a valved device, it regulates the flow, preventing hypotony postoperatively (21). A total of four studies were eligible for inclusion, examining primary and secondary outcomes. No significant difference was seen in IOP; however, at the 2-year interval, there was a substantial difference in the efficacy of the two groups, indicating that the MGI was more effective at controlling IOP at longer intervals. The Molteno implant device can maintain a low IOP more often than the Ahmed implant, most likely because it has been modified to a double-plate model. Two studies in this meta-analysis consisted of double-plate Molteno implants, which provide a larger surface area for the absorption of aqueous humor and hence better IOP control (8, 19). According to the studies, patients with refractory glaucoma can successfully lower their IOP with both AGV and Molteno implants. The Molteno implant requires temporary ligation to achieve a similar effect, whereas the valve in the AGV prevents early postoperative hypotony. Even though the Molteno implant does not have valve-induced resistance, it might allow more aqueous humor to flow to the plate, which could have a definite IOP-lowering effect (7, 22, 23).

The sensitivity analysis of the hypertensive phase revealed that a smaller number of patients with MGIs presented with hypertensive complications. This variance might arise as a result of the immediate aqueous filtration offered by the Ahmed valve, as opposed to the delayed filtration observed with the ligated Molteno implant (20). Nevertheless, one study presented a higher hypertensive phase with the AGV. This discrepancy may be attributed to the different surface areas of the plates employed in the study, with the AGV implant having an intermediate-sized plate and the MGI having a single plate (18).

In a recent meta-analysis, the overall postoperative complication rates were similar between the two implants, the Aurolab aqueous drainage implant and the AGV. However, in our analysis, postoperative complications were fewer in the AGV group than in the MGI group (24).

However, on a larger scale, the AGV is associated with a lower number of overall postoperative complications, and the sensitivity analysis showed that patients with the AGV need fewer antiglaucoma drugs after surgery, demonstrating that it is safer than the MGI. Moreover, the valve mechanism of the AGV reduces the likelihood of hypotony and its related problems. However, the valve may also contribute to long-term issues, such as tube blockage or fibrosis associated with the valve. In contrast, the Molteno implant carries a higher risk of early postoperative problems, such as hypotony, choroidal effusions, and overfiltration, because it lacks a valve. However, Molteno implants may have a lower incidence of delayed problems such as fibrosis or tube blockage (25–27).

However, a few studies suggest that the valve in the AGV controls the flow of aqueous humor, which reduces the risk of hypotony and lessens the need for intensive postoperative medication. In contrast, the Molteno implant might require stricter postoperative treatment plans due to the temporary closure of its tube to prevent excessive filtration in the early stages. As a result, without excessively burdening patients with complex medication schedules, the valve mechanism of the AGV contributes to a more consistent reduction in IOP (25, 28, 29).

The Ahmed implant’s increased safety may be attributable to the restrictiveness of its valve, because, although non-valved devices may be associated with better IOP control, the lack of a restrictive valve that limits the flow amplifies the risk of hypotony and its associated complications (24). In terms of overall outcomes, the success rates for both types of implants are comparable.

This study possesses several notable strengths. Firstly, it conducted a direct comparative analysis between the MGI and the AGV, as opposed to taking an indirect approach. Secondly, in the assessment of the IOP outcome, our meta-analysis segregated the studies based on the duration of the follow-up period. Thirdly, the meta-analysis encompassed studies involving participants afflicted with diverse forms of glaucoma, including refractory, neovascular, or advanced uncontrolled glaucoma.

Furthermore, the scrutiny of the included articles’ quality in our meta-analysis demonstrated the incorporation of reliable studies yielding dependable outcomes. This affirmation ensures the scientific accuracy of the conclusions drawn. Nonetheless, our meta-analysis reveals certain limitations. Firstly, the inclusion of only one RCT highlights the scarcity of relevant RCTs in the literature, potentially introducing selection bias. Incorporating more RCTs could mitigate this concern, yet their limited availability remains a challenge in this field. Secondly, the presence of heterogeneity among the studies raises the potential for publication bias, given the inability to access unpublished results. The modest quantity of included studies and cumulative participant count further underscore the need for caution in generalizing findings. Additionally, the meta-analysis did not explicitly address patients’ comorbidities, such as diabetes, hypertension, cardiovascular diseases, and autoimmune disorders, which could influence surgical outcomes. Moreover, the meta-analysis overlooked the impact of short follow-up periods on long-term insights, omitted a crucial cost-effectiveness assessment, and failed to consider individual patient characteristics, thus affecting the generalizability of results. Lastly, detailed baseline characteristics, including glaucoma severity, were not provided in the included studies (8, 18–20), limiting insights into potential baseline factors influencing outcomes. A comprehensive understanding of these aspects is crucial for refining future research in this field.

## Conclusion

In summary, this meta-analysis assesses the safety and effectiveness of Molteno and Ahmed glaucoma implants, and it indicates that the Molteno implants demonstrate greater success in sustaining low IOP. In contrast, the Ahmed valve exhibits fewer overall complications. Nonetheless, further randomized clinical trials with longer follow-up periods are necessary to validate and refine these conclusions. These trials would not only confirm the existing findings but also enhance our comprehension of the safety profiles of these devices.

## Data availability statement

The original contributions presented in the study are included in the article/**Supplementary Material**. Further inquiries can be directed to the corresponding author.

## Author contributions

AR: Conceptualization, Data curation, Formal Analysis, Investigation, Methodology, Project administration, Software, Supervision, Validation, Writing – original draft, Writing – review & editing. SB: Data curation, Investigation, Methodology, Validation, Writing – original draft, Writing – review & editing. HA: Data curation, Investigation, Project administration, Writing – original draft, Writing – review & editing. MS: Methodology, Project administration, Software, Writing – original draft, Writing – review & editing. AS: Formal Analysis, Investigation, Methodology, Writing – original draft, Writing – review & editing. SR: Data curation, Investigation, Validation, Writing – original draft, Writing – review & editing. BZ: Data curation, Formal Analysis, Writing – original draft, Writing – review & editing. AF: Methodology, Writing – original draft, Writing – review & editing. MA: Formal Analysis, Methodology, Validation, Writing – original draft, Writing – review & editing.
