# Supplementary material for: Meta-analysis of Molteno glaucoma implants and Ahmed glaucoma valves: insights into efficacy and safety for complex glaucoma
Source: Front Ophthalmol (Lausanne). 2023 Dec 11;3:1307439. doi: 10.3389/fopht.2023.1307439 (PMC11182311; doi:10.3389/fopht.2023.1307439)

Supplementary Material

Supplementary Table 1 – Detailed search strategy used in each database.

Supplementary Table 2. Baseline characteristics of the studies

Supplementary Table 3 – Newcastle-Ottawa Quality Assessment for cohort studies.

Supplementary Table 4 – Newcastle-Ottawa Quality Assessment for case-control study.

Supplementary Figure 1 – Risk of bias summary for randomized controlled trial

Supplementary Figure 2 – Risk of bias graph for randomized controlled trial

Supplementary Figure 3 – Leave-one-out analysis plot for intra-ocular pressure

Supplementary Figure 4 – Leave-one-out analysis plot for surgical success rate

Supplementary Figure 5 – Leave-one-out analysis plot for hypertensive phase

Supplementary Figure 6 – Leave-one-out analysis plot for anti-glaucoma medication

Supplementary Table (S1) Detailed search strategy used in each database.

PubMed	(Molteno implant OR ahmed implant and baerveldt implant OR aurolab aqueous drainage implant OR AADI OR aurolab implant) AND (Baerveldt implant OR BGI OR Glaucoma drainage device OR aqueous shunt device) AND (Glaucoma OR High intra-ocular pressure OR High IOP OR Ocular hypertension)
Cochrane Library	Molteno implant OR ahmed implant and baerveldt implant OR aurolab aqueous drainage implant OR AADI OR aurolab implant) AND (Baerveldt implant OR BGI OR Glaucoma drainage device OR aqueous shunt device) AND (Glaucoma OR High intra-ocular pressure OR High IOP OR Ocular hypertension)
Google Scholar	Molteno implant OR ahmed implant and baerveldt implant OR aurolab aqueous drainage implant OR AADI OR aurolab implant) AND (Baerveldt implant OR BGI OR Glaucoma drainage device OR aqueous shunt device) AND (Glaucoma OR High intra-ocular pressure OR High IOP OR Ocular hypertension)
Science Direct	Molteno implant OR ahmed implant and baerveldt implant OR aurolab aqueous drainage implant OR AADI OR aurolab implant) AND (Baerveldt implant OR BGI OR Glaucoma drainage device OR aqueous shunt device) AND (Glaucoma OR High intra-ocular pressure OR High IOP OR Ocular hypertension)

Supplementary Table 2. Baseline characteristics of the studies

Study	Gender		Pre IOP in mmHg (m±SD)		Pre AGM (m±SD)		Follow up (months)		Age (Years)	
	MGI group	AGV group								
	Male / Female	Male / Female	MGI group	AGV group	MGI group	AGV group	MGI group	AGV group	MGI group	AGV group
Taglia et al	NA	NA	33±13	38±16	2.9±1.3	3.7±0.9	NA	NA	NA	NA
Ayyala et al	17/13	14/16	32.7±12.8	32±9	NA	NA	21±8	20±12	68±17	69±19
Yalvac et al	18/9	17/21	39.3±3.9	39.5±4.5	3.4±0.5	3.4±0.5	41.9±17.1	37.0±18.4	58.3±15.4	57.7±10.9
Nassiri et al	22/24	25/21	33.06±1.66	30.81±1.67	2.7±0.11	2.8±0.11	NA	NA	63.3±1.62	59.4±1.51

(MGI: Molteno Glaucoma Implant; AGV: Ahmed Glaucoma Valve; Pre IOP: preoperative intraocular pressure; Pre AGM: preoperative antiglaucoma medication; (m±SD): mean and standard deviation; NA: Not Available

Supplementary Table (S3) Newcastle-Ottawa quality assessment scale for cohort studies

	**Study name**	
	Taglia 2002	Yalvac 2005
**Selection (4)**		
Representativeness of the exposed cohort	*	*
Selection of the non-exposed cohort	*	*
Ascertainment of exposure	*	*
Demonstration that outcome of interest was not present at start of study	*	*
**Comparability (2)**		
Comparability of cohorts on the basis of the design or analysis	**	**
**Outcome (3)**		
Assessment of outcome		*
Was follow-up long enough for outcomes to occur	*	*
Adequacy of follow up of cohorts	*	*
**Total (9)**	**8**	**9**

Supplementary Table (S4) Modified Newcastle-Ottawa quality assessment scale for case-control study

	**Study name**
	Ayyala 2002
**Selection (4)**	
Is the case definition adequate?	*
Representativeness of the cases	*
Selection of Controls	*
Definition of Controls	*
**Comparability (2)**	
Comparability of cases and controls on the basis of the design or analysis	**
**Exposure (3)**	
Ascertainment of exposure	
Same method of ascertainment for cases and controls	
Non-Response rate	*
**Total (9)**	**7**

Supplementary Figure (S1) Risk of bias summary for randomized controlled trial

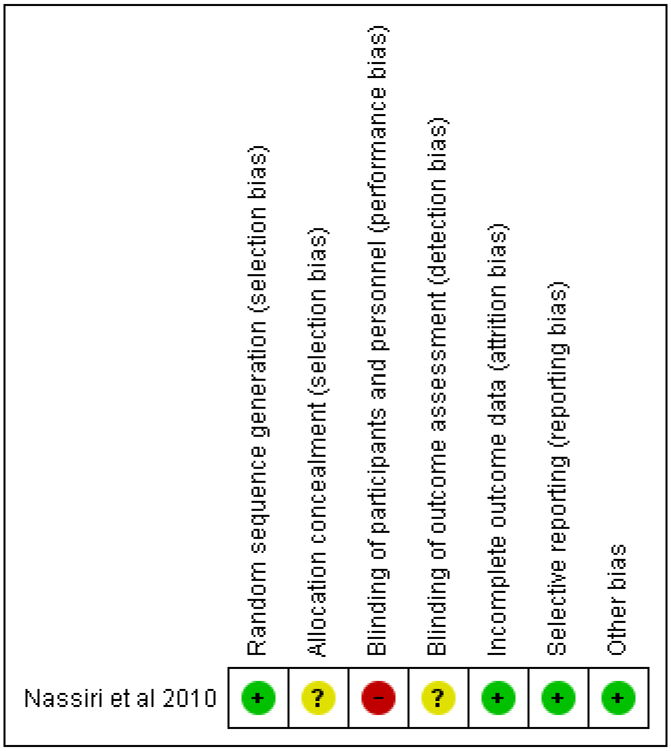

Supplementary Figure (S2) Risk of bias graph for randomized controlled trial

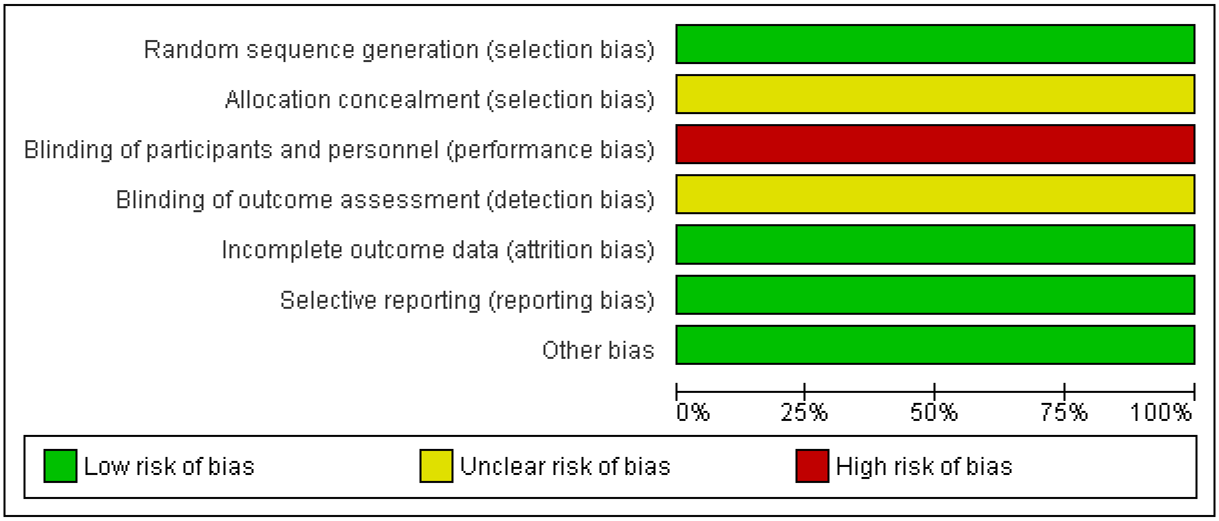

Supplementary Figure (S3). Leave-one-out analysis for intra-ocular pressure

	Molteno Glaucoma Implant			Ahmed Glaucoma Valve				Mean Difference
**Study or Subgroup**	**Mean**	**SD**	**Total**	**Mean**	**SD**	**Total**	**Weight**	**IV, Random, 95% CI**
**3.1.1 Post operative IOP at 3 months**								
Ayyala et al 2002	15.1	7.4	30	19.5	6.5	30	8.1%	-4.40 [-7.92, -0.88]
Yalvac et al 2005	21.3	9.6	27	15.6	8	38	0.0%	5.70 [1.27, 10.13]
**Subtotal (95% CI)**			**30**			**30**	**8.1%**	**-4.40 [-7.92, -0.88]**
Heterogeneity: Not applicable								
Test for overall effect: Z = 2.45 (P = 0.01)								
**3.1.2 Post operative IOP at 6 months**								
Ayyala et al 2002	14.6	8.8	30	17.8	7.2	30	6.5%	-3.20 [-7.27, 0.87]
Yalvac et al 2005	20.9	6.8	27	17.8	7.2	38	0.0%	3.10 [-0.34, 6.54]
**Subtotal (95% CI)**			**30**			**30**	**6.5%**	**-3.20 [-7.27, 0.87]**
Heterogeneity: Not applicable								
Test for overall effect: Z = 1.54 (P = 0.12)								
**3.1.3 Post operative IOP at 1 year**								
Ayyala et al 2002	13.3	5.2	20	16.7	5.6	30	9.9%	-3.40 [-6.43, -0.37]
Taglia et al 2002	13.8	4.8	27	17.4	4.5	13	9.9%	-3.60 [-6.64, -0.56]
Yalvac et al 2005	20.8	7.7	27	19.7	6.7	38	7.8%	1.10 [-2.50, 4.70]
**Subtotal (95% CI)**			**74**			**81**	**27.6%**	**-2.12 [-4.94, 0.71]**
Heterogeneity: Tau² = 3.54; Chi² = 4.63, df = 2 (P = 0.10); I² = 57%								
Test for overall effect: Z = 1.47 (P = 0.14)								
**3.1.4 Post operative IOP at 2 year**								
Ayyala et al 2002	13.3	5.1	16	19	5.8	13	6.6%	-5.70 [-9.72, -1.68]
Nassiri et al 2010	15.36	0.33	46	17	0.23	46	28.6%	-1.64 [-1.76, -1.52]
Yalvac et al 2005	21	6.5	27	21.5	6.9	38	8.9%	-0.50 [-3.79, 2.79]
**Subtotal (95% CI)**			**89**			**97**	**44.1%**	**-2.12 [-4.23, -0.01]**
Heterogeneity: Tau² = 2.01; Chi² = 4.37, df = 2 (P = 0.11); I² = 54%								
Test for overall effect: Z = 1.97 (P = 0.05)								
**3.1.5 Post operative IOP at 3 year**								
Ayyala et al 2002	13	9	8	18.6	5.1	10	2.6%	-5.60 [-12.59, 1.39]
Yalvac et al 2005	22.2	5.4	27	21.6	5.9	38	11.1%	0.60 [-2.17, 3.37]
**Subtotal (95% CI)**			**35**			**48**	**13.7%**	**-1.63 [-7.47, 4.20]**
Heterogeneity: Tau² = 11.86; Chi² = 2.61, df = 1 (P = 0.11); I² = 62%								
Test for overall effect: Z = 0.55 (P = 0.58)								
**Total (95% CI)**			**258**			**286**	**100.0%**	**-2.14 [-3.32, -0.96]**
Heterogeneity: Tau² = 1.26; Chi² = 16.14, df = 9 (P = 0.06); I² = 44%								
Test for overall effect: Z = 3.55 (P = 0.0004)								
Test for subgroup differences: Chi² = 1.50, df = 4 (P = 0.83), I² = 0%								

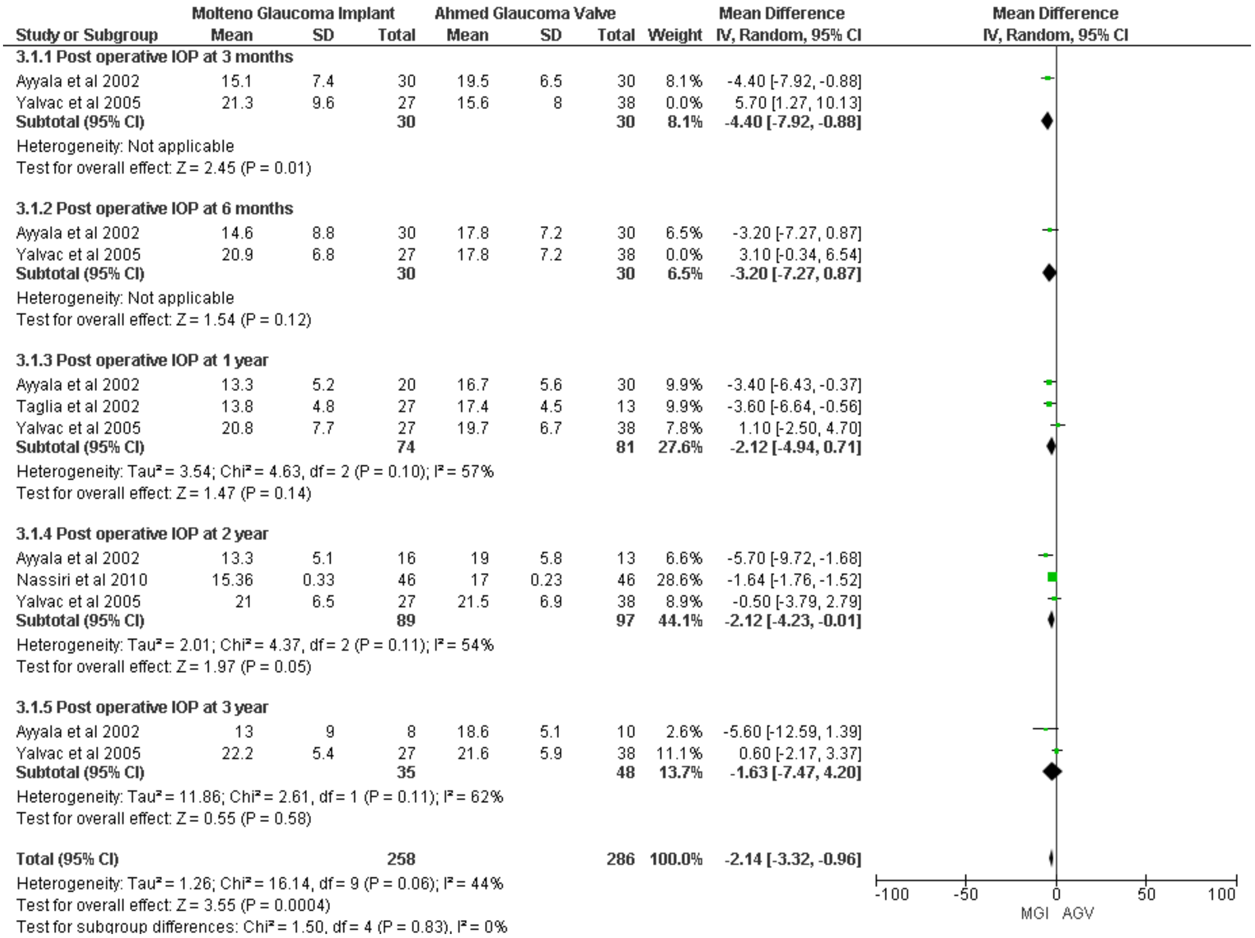

Supplementary Figure (S4). Leave-one-out analysis for surgical success rate

	Molteno Glaucoma Implant		Ahmed Glaucoma Valve			Risk Ratio	
Study or Subgroup	Events	Total	Events	Total	Weight	M-H, Random, 95% CI	Year
Ayyala et al 2002	15	30	17	30	38.3%	0.88 [0.55, 1.42]	2002
Taglia et al 2002	15	27	3	13	11.8%	2.41 [0.84, 6.87]	2002
Yalvac et al 2005	7	27	20	28	0.0%	0.36 [0.18, 0.72]	2005
Nassiri et al 2010	19	28	19	29	49.9%	1.04 [0.72, 1.49]	2010
**Total (95% CI)**		**85**		**72**	**100.0%**	**1.08 [0.73, 1.58]**	
Total events	49		39				

Heterogeneity: Tau² = 0.04; Chi² = 3.13, df = 2 (P = 0.21); I² = 36%
Test for overall effect: Z = 0.37 (P = 0.71)

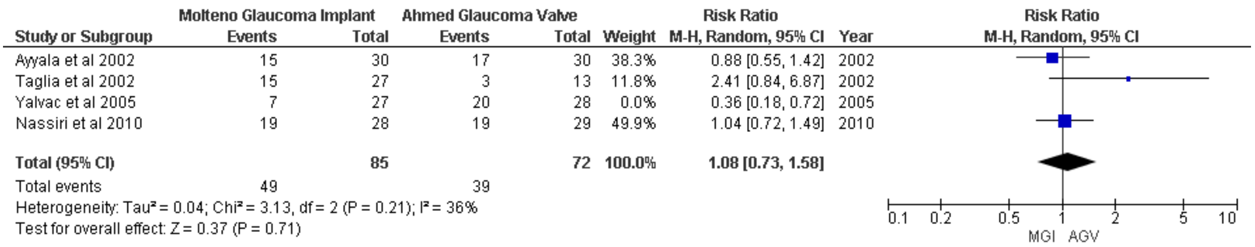

Supplementary Figure (S5). Leave-one-out analysis for hypertensive phase

	Molteno Glaucoma Implant		Ahmed Glaucoma Valve			Risk Ratio	
Study or Subgroup	Events	Total	Events	Total	Weight	M-H, Random, 95% CI	Year
Ayyala et al 2002	13	30	25	30	75.9%	0.52 [0.34, 0.81]	2002
Yalvac et al 2005	8	27	7	38	0.0%	1.61 [0.66, 3.90]	2005
Nassiri et al 2010	8	46	13	46	24.1%	0.62 [0.28, 1.34]	2010
**Total (95% CI)**		**76**		**76**	**100.0%**	**0.54 [0.37, 0.79]**	
Total events	21		38				

Heterogeneity: Tau² = 0.00; Chi² = 0.15, df = 1 (P = 0.70); I² = 0%
Test for overall effect: Z = 3.14 (P = 0.002)

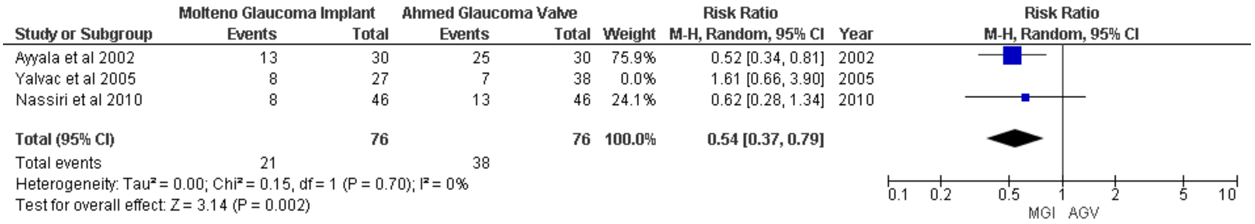

Supplementary Figure (S6). Leave-one-out analysis for anti-glaucoma medication

	Molteno Glaucoma Implant			Ahmed Glaucoma Valve				Mean Difference	
Study or Subgroup	Mean	SD	Total	Mean	SD	Total	Weight	IV, Random, 95% CI	Year
Taglia et al 2002	0.48	0.87	27	1.5	1.51	13	0.0%	-1.02 [-1.90, -0.14]	2002
Yalvac et al 2005	1.8	1.2	27	1.7	1.6	38	1.9%	0.10 [-0.58, 0.78]	2005
Nassiri et al 2010	1.41	0.19	46	1.03	0.27	46	98.1%	0.38 [0.28, 0.48]	2010
**Total (95% CI)**			**73**			**84**	**100.0%**	**0.37 [0.28, 0.47]**	

Heterogeneity: Tau² = 0.00; Chi² = 0.64, df = 1 (P = 0.42); I² = 0%
Test for overall effect: Z = 7.77 (P < 0.00001)

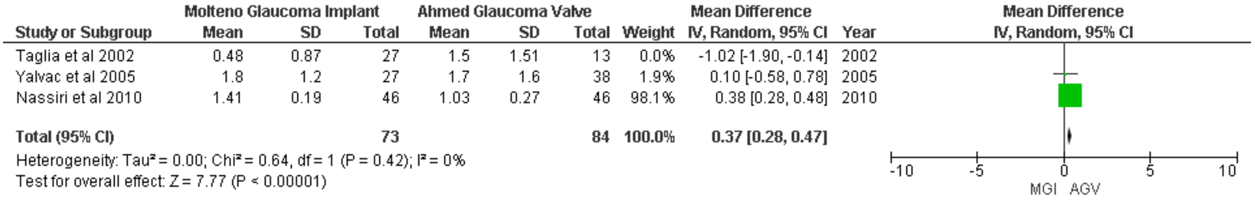

Supplement: Supplementary file 1 [file DataSheet_1.pdf]
